# Insights on the spatial distribution of the *Pseudomonas aeruginosa* secondary metabolites under swarming motility-inducing conditions using mass spectrometry imaging

**DOI:** 10.1128/spectrum.01368-25

**Published:** 2025-11-11

**Authors:** Joenisse M. Rosado-Rosa, Dharmeshkumar Parmar, Joshua D. Shrout, Jonathan V. Sweedler

**Affiliations:** 1 Department of Chemistry and Beckman Institute for Advanced Science and Technology, University of Illinois Urbana-Champaign 14589, Urbana, Illinois, USA; 2 Department of Civil and Environmental Engineering and Earth Sciences, University of Notre Dame 6111, Notre Dame, Indiana, USA; 3 Department of Biological Sciences, University of Notre Dame 6111, Notre Dame, Indiana, USA; Emory University School of Medicine, Atlanta, Georgia, USA

**Keywords:** tendrils, pyocyanin, quinolones, spatiochemical, metabolomics

## Abstract

*Pseudomonas aeruginosa* exhibits a variety of motility types, including twitching, swimming, and swarming motility. The latter is typically associated with growth on semi-solid surfaces (e.g., 0.4% to 0.5% agar), the formation of extending tendrils, and the secretion of rhamnolipids, a biosurfactant that aids cells in spreading along a surface. Apart from rhamnolipids, *P. aeruginosa* also secretes a variety of secondary metabolites involved in pathogenesis and defense. We developed a mass spectrometry imaging (MSI) workflow to characterize metabolites associated with swarming motility in *P. aeruginosa* and evaluated how externally applied rhamnolipids influence swarming behavior and secondary metabolite production. MSI allowed us to measure spatial profiles of prominent rhamnolipid congeners and secondary metabolites. The addition of rhamnolipids over a broad concentration range (0.0001 to 1 mg/mL) into swarming media prior to inoculation allowed for the study of concentration-dependent effects of rhamnolipids, as well as changes in the secretion profile. Unlike self-produced rhamnolipids that facilitate swarming, externally applied rhamnolipids significantly reduced swarming behavior even at low concentrations. This suggests a potential reason why tendrils from swarming cultures avoid overlap. Insights on the differential distribution of secondary metabolites within these bacterial communities on agar improve our understanding of biofilm development. The use of MSI on *P. aeruginosa* swarming cultures provides detailed spatial chemical analyses of the secreted molecules under varying conditions.

**IMPORTANCE:**

The semi-solid surface that encourages *Pseudomonas aeruginosa* to exhibit swarming motility has a similar consistency to a stationary, thickened gel commonly associated with impaired lung surfaces during infection. The effects rhamnolipids have on the spreading properties of *P. aeruginosa* under these conditions have yet to be elucidated. Here, we used mass spectrometry imaging to probe the spatial-chemical profiles of the surface to provide a more complete and unbiased understanding of the distribution of important classes of molecules secreted by *P. aeruginosa,* including rhamnolipids and a range of secondary metabolites on surfaces. The application of rhamnolipids prior to inoculation allows us to understand how these molecules affect the motility seen under moist, semi-solid conditions.

## INTRODUCTION

In the laboratory, *Pseudomonas aeruginosa* swarming is seen under low agar (0.4% to 0.5%) conditions, generating a spreading pattern with distinctive tendrils that aid surface colonization (1). *In vivo*, *P. aeruginosa* swarming is perceived to occur under moist conditions, such as those found within lungs and skin lesions (2–4). Swarming motility is a significant behavior as it offers the fastest mode of translocation while ensuring survival at high cell density (5, 6). Studies investigating swarming-deficient phenotypes show reduced growth and colonization in skin infection models, highlighting the importance of swarming for *P. aeruginosa in vivo* (4). Swarming requires functional flagella as well as active quorum sensing to produce the biosurfactants known as rhamnolipids (7–9).

The rhamnolipids secreted by *P. aeruginosa* modulate swarming motility by reducing liquid surface tension to allow movement for large numbers of cells (10, 11). These biosurfactants are comprised of a rhamnose moiety and a fatty acid moiety. *P. aeruginosa* can produce over 28 rhamnolipid congeners which are divided into four types based on the number of rhamnose and lipid moieties: mono-rhamnose-mono-lipid (R_1_L_1_), mono-rhamnose-di-lipid (R_1_L_2_), di-rhamnose-mono-lipid (R_2_L_1_), and di-rhamnose-di-lipid (R_2_L_2_) (12). They have been identified and/or characterized through cell stain imaging techniques and LC-MS (13, 14). Although rhamnolipids are typically associated with swarming, how different levels of rhamnolipid abundance influence motility and other mechanisms, such as quorum sensing and virulence factor production, remains unknown.

In the multi-layered hierarchy of quorum sensing networks that *P. aeruginosa* follows, alkyl-4-quinolones are strongly associated with the *Pseudomonas* genus and not other Gram-negative bacteria that also utilize acyl homoserine lactone quorum sensing signals. The most abundant quinolone in *P. aeruginosa* is often 2-heptyl-3-hydroxy-4-quinolone or *Pseudomonas* quinolone signal (PQS) (15), which has been directly correlated to biofilm regulation through the mediation of the LysR-type transcriptional regulator PqsR (16). Abundance of PQS and other quinolones can also modulate swarming motility (17–19). PQS is also a quorum sensing signal for the PQS regulon that can upregulate production of phenazines, a group of redox-active compounds, such as pyocyanin (PYO). PYO is important for virulence and survival of *P. aeruginosa* infection and is well-studied (20, 21). PYO has been correlated to oxidative stress, cell toxicity, and biofilm formation (22), and is detectable in *P. aeruginosa* swarms (23).

These sets of secondary metabolites are secreted during infection and have been found in lung sputum from CF patients during *P. aeruginosa* infections (2, 3, 24–27). The role of some of these molecules during infection has been elucidated; however, the function of others remains to be assigned. Identifying the spatial location of these molecules could lead to a better understanding of their potential roles during infection. Identification and characterization of these molecules have been performed using analytical and biomolecular methods, such as chromatography (TLC, HPLC-UV, GC, LC-MS), mass spectrometry (matrix-assisted laser desorption ionization [MALDI], ESI, LC), Raman microscopy, genetic reporters, and staining dyes (3, 12, 14, 16, 24, 28–30). These metabolites are typically extracted from a *P. aeruginosa* culture for further analysis or are tagged using transcriptomics and molecular biology techniques, which can be extensive techniques and require long preparation or data collection.

Mass spectrometry imaging (MSI) is a rapidly emerging tool for spatial chemical characterization of biological samples. MSI allows for the identification of a wide range of molecules on a two-dimensional sample surface while linking the information to spatial coordinates. Thus, MSI enables us to visualize the localization of molecules in the form of ion images. MSI has helped elucidate a range of effects, properties, and spatial distribution of relevant metabolites, lipids, and peptides from colonies and biofilms of several bacteria, including *P. aeruginosa* (30–34). This includes agar cultures, which can be analyzed whole through MSI. Swarming is only observed on a semi-solid surface, which makes MSI a useful tool for identifying secreted secondary metabolites in *P. aeruginosa* swarm cultures on solid surfaces.

MALDI and secondary ion mass spectrometry (SIMS) are the frequently reported surface ionization techniques for MSI analysis (31). *P. aeruginosa* MSI swarming studies have previously been reported by our laboratory (35–37) to identify the spatial localization of PQS and alkyl quinolone metabolites using SIMS and confocal Raman microscopy (CRM). Used in tandem, MSI and CRM can aid in the identification of biomolecules in bacterial cultures (35). During these studies, changes in the environment were made involving antibiotic addition (36) or the growth of a secondary *P. aeruginosa* strain, FRD1 (37), to the growth media to observe environmental and molecular changes. The identification of quinolone metabolites spatially distributed on the bacterial surface is observed throughout the bacterial growth surface (37). A putative rhamnolipid with a mass of 525.26 m/z was identified in the MSI culture as well, although no other rhamnolipid or other secondary metabolites were reported. MSI analysis collected during these studies lacks further information on the spatiochemical characterization of other *P. aeruginosa* secondary metabolites often associated with swarming and biofilm formation, like rhamnolipids and phenazines. Prior to these MSI studies, rhamnolipids on swarming cultures were identified using stains (14), which limits the identification of the rhamnolipid congeners as the chosen dye stains general lipid moieties. Further improvements on the sample preparation to analyze swarming cultures and the tendrils formed during the process can enhance our understanding of additional analytes that can serve as behavioral biomarkers of *P. aeruginosa* swarming motility outside of PQS and alkyl quinolones. Although these studies resulted in insightful information about *P. aeruginosa* behavior, here we enhance and simplify these approaches. As swarming cultures are performed using low agar concentrations, the preparation and transfer of the agar cultures are important to prevent perturbation of the colony. Large swarm colonies (>1 cm) tend to be more difficult to transfer due to the fragility of the agar at that size. Previous studies have performed MSI analysis of smaller swarm cultures (<0.15 cm) (35, 36). A streamlined method must be established for MSI sample preparation of larger swarming cultures to facilitate detailed spatiochemical profiling of *P. aeruginosa* under swarming conditions.

For this study, we developed an optimized sample preparation workflow for MSI analysis of *P. aeruginosa* swarming cultures, which we used MALDI-MSI to identify and locate secondary metabolites in swarming *P. aeruginosa* cultures. The use of a new generation of instrumentation such as the timsTOF fleX MALDI-2 allows for an enhanced MS image, where distinct secondary metabolites are identified throughout a swarming *P. aeruginosa* culture. The method development is detailed for sample reproducibility in future MSI experiments and studies, as the analytical workflow for MSI swarm studies has yet to be established. The MSI analysis allowed us to identify the spatial distribution of important secondary metabolites from the quinolone, phenazine, and rhamnolipid classes, the last two of which have yet to be reported on MSI studies of *P. aeruginosa* swarming cultures. The subsequent addition of a rhamnolipid mixture to the media prior to inoculation, followed by MSI analysis, delineated the effects of biosurfactants on the growth and localization of secondary metabolites during swarming. This study demonstrates that MSI is a valuable tool for examining swarming motility and the influence of endogenous metabolites on bacterial behavior.

## RESULTS

### Swarming media optimization for MSI analysis

**Fig 1:**
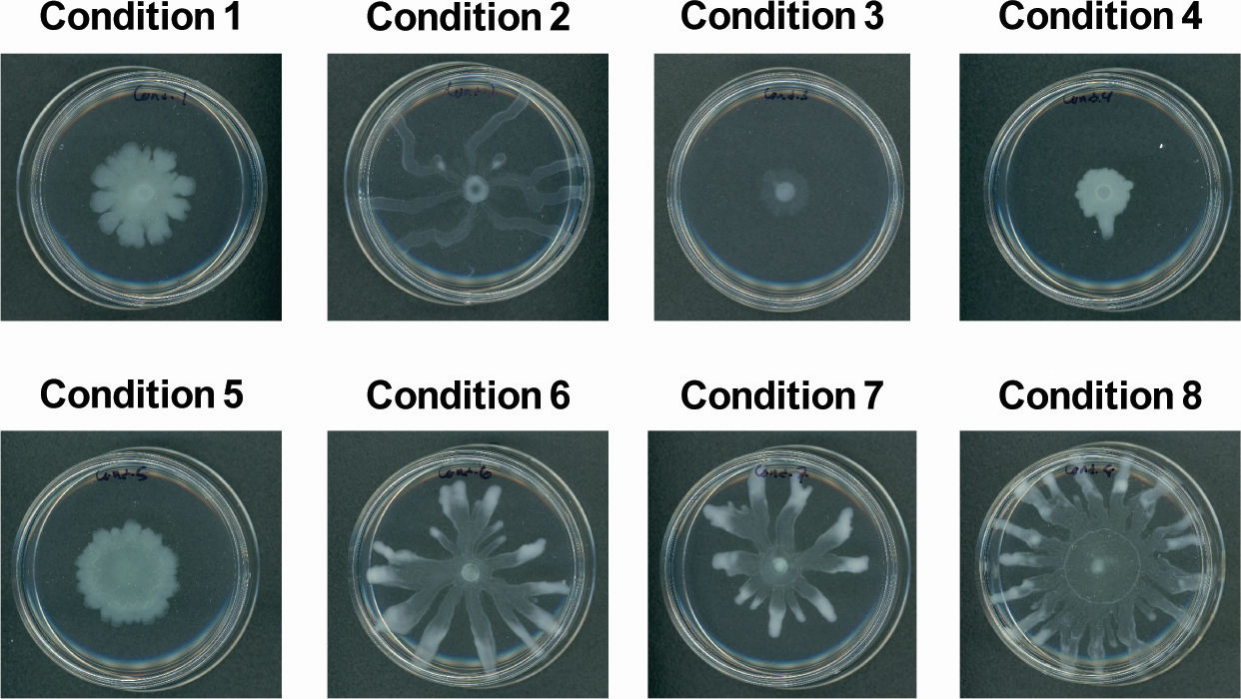
M8 minimal media swarming condition test using different chemical concentrations listed in Table 1. Condition 1 is the condition that produces tendrils with a uniform cell density. This was chosen as the condition of choice for this study. Petri dish size used in all conditions is 60 × 15 mm.

*P. aeruginosa* swarms under a range of different media conditions, including M8 minimal media, M9 minimal media, BM2 minimal media, and Luria-Bertani (LB), to name a few (23, 38–40). Here, we used M8 minimal medium as it is a popular and convenient medium to induce swarming and, thus, of relevance for future investigations. An ideal tendril morphology includes spatially well-separated tendrils so that they can be analyzed via MSI. Starting with M8 minimal medium, we tested a number of modifications, including different concentrations of MgSO_4_, CaCl_2_, glucose, and Casamino acids (Table 1). For the purposes of reproducibility and method development, we used three criteria to choose the conditions to use for MSI analysis. These criteria involve symmetry for MSI data reproducibility, tendril formation to confirm swarming, and uniform color suggesting an even distribution of metabolites throughout the culture. Of all eight conditions tested, condition 1 stood out due to its swarming features, following desired criteria of tendril production and uniform cell density (Fig. 1). Notable observations from all the conditions tested (Fig. 1) include several with a lack of growth (conditions 3 and 4), a lack of tendril formation (condition 5), and discoloration into a translucent growth throughout or in the inner spread of the tendrils (conditions 2, 3, and 6–8). Although condition 8 had distinct tendrils and seemed to be a good candidate for MSI studies, the subsequent application of chemicals for behavioral studies had no effect on the tendril growth, contradicting results observed on the other conditions tested (conditions 1–7; data not shown). Therefore, we used the media parameters for condition 1 for follow-up MSI swarming experiments. Compared to the other conditions mentioned above (BM2 minimal media, LB, M9 minimal media), the M8 minimal media conditions allow for symmetrical reproducibility of the tendrils, where all grown cultures under the same condition allow all cultures prepared to resemble each other allowing us to analyze the culture through MSI, as the definition of tendrils is of interest to better understand the chemical profile of *P. aeruginosa*.

**TABLE 1:** M8 minimal medium recipe conditions used in this study^*a*^

Condition	M8 salts	MgSO_4_	CaCl_2_	Glucose	Casamino acids	Reference
1	1×	1 mM	–^*b*^	0.2%	0.5%	(10)
2	1×	1 mM	0.1 mM	0.5%	0.1%	(41)
3	1×	2 mM	–^*b*^	0.2%	0.05%	(23)
4	1×	1 mM	0.1 mM	0.2%	0.5%	This study
5	1×	1 mM	1 mM	0.2%	0.5%	This study—modeled by the M9 recipe (42)
6	1×	1 mM	–^*b*^	0.5%	0.5%	This study
7	1×	1 mM	0.1 mM	0.5%	0.5%	This study
8	1×	1 mM	1 mM	0.5%	0.5%	This study—modeled by the M9 recipe (42)

^*a*^ M8 is composed of 48 mM Na_2_HPO_4_, 24 mM KH_2_PO_4_, and 8 mM NaCl. All added chemicals were diluted from a stock: 10× M8 salts, 500 mM MgSO_4_, 500 mM CaCl_2_, 50% glucose, and 20% Casamino acids.

^*b*^ ”–” indicates the absence of chemical in the media.

**Fig 2:**
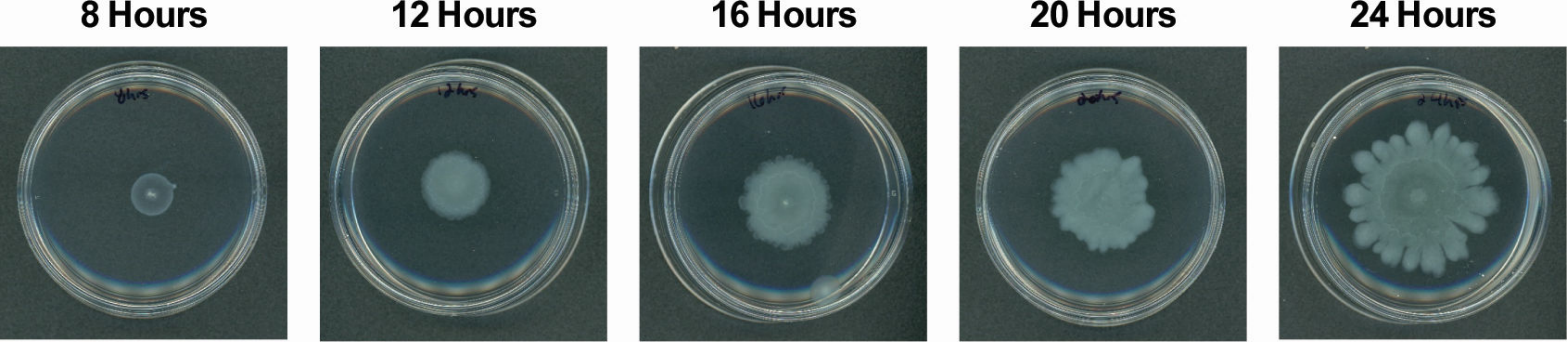
Swarming culture growth studies. Tendrils start to form between 20 and 24 h; 18 h was chosen as the incubation time for all cultures in the study to allow an extra 4–6 h of growth while drying at 37°C. Petri dish size used in all conditions is 60 × 15 mm.

Our method to read out spatiochemical information from swarming cultures is through MALDI-MSI. MALDI MS has a distinct set of sample requirements and so requires a different set of optimizations. For example, MALDI-MSI is performed under vacuum conditions and, therefore, requires dehydrated samples. In order to dehydrate the samples, agar is transferred to a MALDI target plate. In addition, agar drying time must be considered as it adds time for the culture to grow beyond what is needed for MSI analysis. Therefore, optimizing incubation time is important in this regard due to two factors: (i) growth time affects the molecules produced in the culture or could change the morphology due to overgrowth and (ii) sample drying requires time that can affect said production and give different results. Although sample drying can come in many forms, two of the most popular drying methods for bacterial cultures are forced air and heat (32, 43–45). After testing both, we found that heat gave uniform drying compared to forced air, which dried the agar unevenly. Forced air led to loss of signal on the sample and flaking and/or cracking after prolonged drying (data not shown). All samples were then dried using heat by incubating the samples at 37°C post-transfer into the MALDI target plate. To prepare the samples for MSI, we transferred the whole agar assays from their Petri dishes onto a MALDI target plate for drying. Agar transfer was performed carefully as the agar is fragile at such a low agar concentration and can easily break. Typical transfer involves breaking the edges of the Petri dish and sliding the agar onto the target plate by tilting the dish at an angle and carefully pushing it with a spatula. Attempts to trim unneeded edges of the agar must be performed post transfer to the target plate as it could not only perturb the culture but also damage the agar, which causes breakage during transfer. Lifting of the agar also causes breakage and must be avoided. The target plates were allowed to dry at 37°C for 4–6 h. Since the cultures are further incubated at 37°C past the growth time, we assessed the optimal incubation time to prevent culture overgrowth during the drying phase. Growth time points were taken every 4 h starting from 8 h until 24 h. In Fig. 2, optical images of swarming cultures at these time points can be observed. Tendrils start to form around 16 h under these conditions. At 24 h, clearly distinct tendrils are observed. Thus, growing swarming cultures until 18–20 h prior to drying and then further incubating them to dry the cultures will prevent overgrowth while still having distinct tendrils for MSI analysis.

### MSI analysis of swarming cultures in semi-solid and solid conditions

**Fig 3:**
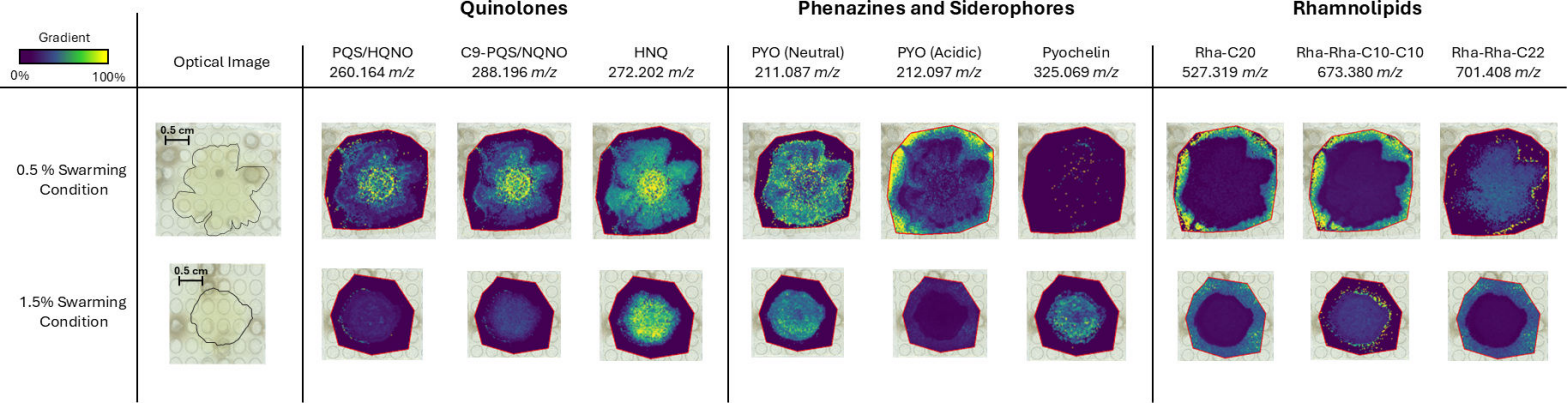
MSI analysis of 0.5% swarming and 1.5% agar conditions. The yellow color indicates higher chemical abundance, while the blue color indicates lower chemical abundance in the image. Accumulation of signal outside the delineated area on the optical image corresponds to signal observed on the uncolonized agar surface. Scale bars (0.5 cm) can be found in the optical image of each condition.

As *P. aeruginosa* swarming is observed in moist, semi-solid conditions, we prepared a 1.5% agar M8 medium control that should yield standard bacterial colonies to compare against the 0.5% swarming assays. This allows us to identify changes in secondary metabolites associated with swarming as compared to the compounds in the media and agar. All conditions were performed in triplicate (Fig. S1), and a replicate was chosen for MSI analysis as a representative of the batch. After the colony is dried, MALDI-MSI requires the application of a matrix that facilitates the ionization and detection of the metabolites. All culture conditions were analyzed through MSI. A list of molecules detected through MSI can be found in Tables 2 to 4. Figure 3 shows MSI images corresponding to some of the secreted molecules found in Tables 2 to 4, chosen by their importance in their corresponding quinolone, phenazine, or rhamnolipid families and their higher abundance as observed using MSI. Notable differences in the molecules' intensity between the prepared cultures can be observed for the biofilm-related PQS/HQNO and C9-PQS/NQNO quinolones, the acidic PYO and pyochelin phenazines, and the Rha-Rha-C10-C10 and Rha-Rha-C22 rhamnolipids (Fig. 3). Interestingly, we observed the spatial distribution of two rhamnolipids (Rha-C20 and Rha-Rha-C10-C10) outside of the *P. aeruginosa* swarming culture on the agar surface, indicating that these rhamnolipids diffuse away from the colony onto the agar surface during bacterial expansion. The three quinolone molecules are localized in the center of the culture, denoted by the yellow color, indicating that these molecules aggregate in the inner part of the culture over the tendrils. PYO has been identified in two forms in *P. aeruginosa* (46). Under acidic conditions (pH <5), PYO is reduced and is used as a defense mechanism against invading organisms; meanwhile, under neutral or basic conditions, PYO is oxidized and can generate oxidative stress through the production of reactive oxygen species and can easily penetrate the cell membrane (46). Both PYO forms are observed in test conditions (Fig. 3). However, while the neutral PYO is exclusively observed within the colonized areas, the acidic PYO is observed at higher abundance in the swarming culture and is distinctly distributed beyond the colonized region.

**TABLE 2:** Quinolone metabolites observed in MSI; quinolones not observed in the MSI data appeared as blank images

Quinolones	Abbreviation	Molecular formula	Adduct	Calculated m/z	Observed m/z
2-Methyl-3-hydroxy-4-quinolone	MPQS	C_10_H_9_NO_2_	[M + H]^+^	176.07	176.07
2-Propyl-3-hydroxy-4-quinolone	C3-PQS	C_12_H_13_NO_2_	[M + H]^+^	204.10	204.10
Quinolobactin	–^*a*^	C_11_H_9_NO_4_	[M + H]^+^	220.06	Not observed
2-Pentyl-3-hydroxy-4-quinolone	C5-PQS	C_14_H_17_NO_2_	[M + H]^+^	232.14	Not observed
2-Heptyl-4(1H)-quinolone	HHQ	C_16_H_21_NO	[M + H]^+^	244.17	244.17
2-Heptyl-3-hydroxy-4-quinolone	PQS	C_16_H_21_NO_2_	[M + H]^+^	260.17	260.17
2-Heptyl-4-quinoline N-oxide	HQNO	C_16_H_21_NO_2_	[M + H]^+^	260.17	260.17
2-Nonyl-4(1H)-quinolone	HNQ	C_18_H_25_NO	[M + H]^+^	272.20	272.20
2-Nonyl-3-hydroxy-4-quinolone	C9-PQS	C_18_H_25_NO_2_	[M + H]^+^	288.20	288.20
2-Nonyl-4-quinoline N-oxide	NQNO	C_18_H_25_NO_2_	[M + H]^+^	288.20	288.20

^*a*^ –, not available.

**TABLE 3:** Phenazine metabolites observed in MSI; phenazines not observed in the MSI data appeared as blank images

Phenazines	Abbreviation	Molecular formula	Adduct	Calculated m/z	Observed m/z
Phenazine	PHZ	C_12_H_8_N_2_	[M + H]^+^	181.08	181.08
1-Hydroxyphenazine	1OH-PHZ	C_12_H_8_N_2_O	[M + H]^+^	197.07	197.07
2-Hydroxyphenazine	2OH-PHZ	C_12_H_8_N_2_O	[M + H]^+^	197.07	197.0
Neutral pyocyanin	PYO (Neut.)	C_13_H_10_N_2_O	[M + H]^+^	211.09	211.09
Acidic pyocyanin	PYO (Ac.)	C_13_H_11_N_2_O^+^	[M + H]^+^	212.10	212.10
Phenazine-1-carboxamide	PCN	C_13_H_9_N_3_O	[M + H]^+^	224.08	Not observed
Phenazine-1-carboxylic acid	PCA	C_13_H_8_N_2_O_2_	[M + H]^+^	225.07	225.07
5-Methyl-phenazinium-1-carboxyate	5MPCA	C_14_H_11_N_2_O_2_^+^	[M + H]^+^	240.09	Not observed
2-Hydroxyphenazine-1-carboxylic acid	2OH-PCA	C_13_H_8_N_2_O_3_	[M + H]^+^	241.06	Not observed
Phenazine-1,6-dicarboxylic acid	PDC	C_14_H_8_N_2_O_4_	[M + H]^+^	269.06	Not observed

**TABLE 4:** Rhamnolipid congeners observed in MSI; rhamnolipids not observed in the MSI data appeared as blank images^*a*^

Rhamnolipid	Rhamnolipid type	Molecular formula	Isomers	Adduct	Calculated m/z	Observed m/z
Rha-C10	R_1_L_1_	C_16_H_30_O_7_	N/A	[M + Na]^+^	357.19	357.19
Rha-C12:2	R_1_L_1_	C_18_H_30_O_7_	N/A	[M + Na]^+^	381.19	381.19
Rha-C14:2	R_1_L_1_	C_20_H_34_O_7_	N/A	[M + Na]^+^	409.22	409.22
Rha-C18	R_1_L_2_	C_24_H_44_O_9_	2	[M + Na]^+^	499.29	Not observed
Rha-C20	R_1_L_2_	C_26_H_48_O_9_	3	[M + Na]^+^	527.32	527.32
Rha-C22	R_1_L_2_	C_28_H_52_O_9_	2	[M + Na]^+^	555.35	Not observed
Rha-Rha-C10	R_2_L_1_	C_22_H_40_O_11_	N/A	[M + Na]^+^	503.25	503.25
Rha-Rha-C8-C8	R_2_L_2_	C_28_H_50_O_13_	N/A	[M + Na]^+^	617.31	617.31
Rha-Rha-C10-C10	R_2_L_2_	C_32_H_58_O_13_	N/A	[M + Na]^+^	673.38	673.38
Rha-Rha-C22	R_2_L_2_	C_34_H_62_O_13_	2	[M + Na]^+^	701.41	701.41

^*a*^ The number of isomers indicates the different species with equal m/z for the congeners. The isomers could not be distinguished from each other, so they were reported together.

In contrast with the swarming culture (0.5% M8 media), the 1.5% agar M8 medium culturing condition lacks the abundance of PQS/HQNO and C9-PQS/NQNO, as well as acidic PYO (Fig. 3). Pyochelin, a siderophore produced by *P. aeruginosa* (47), is evenly distributed throughout the 1.5% agar colonies, while it is not observed under 0.5% agar semi-solid swarming conditions. Additionally, MSI analysis also revealed the distribution profiles of rhamnolipids and differences between swarming and non-swarming cultures. Further, each congener shows distinct differences between the two culture conditions. The rhamnolipid congeners, Rha-C20 and Rha-Rha-C10-C10, were the predominant rhamnolipids observed in swarming cultures. Both were also localized on the agar surface, outlining the tendrils and the space beyond on the surface. This was not observed in the condition with 1.5% agar. Contrary to that, Rha-Rha-C22 was detected mostly within the colonized region under swarming conditions and outside in 1.5% agar. Ion images generated from swarming and non-swarming colonies allowed us to identify specific chemical changes associated with swarming conditions. The modulation observed here opens a promising avenue to explore bacterial swarming and highlights the use of MALDI-MSI as a tool to study how metabolic pathways in *P. aeruginosa* are modulated during swarming behavior. Specifically, the data indicate changes in localized abundance of quinolones, phenazines, and rhamnolipids under relatively small changes in culturing conditions.

### Environmental changes in swarming conditions change the secretion profile of *P. aeruginosa*

**Fig 4:**
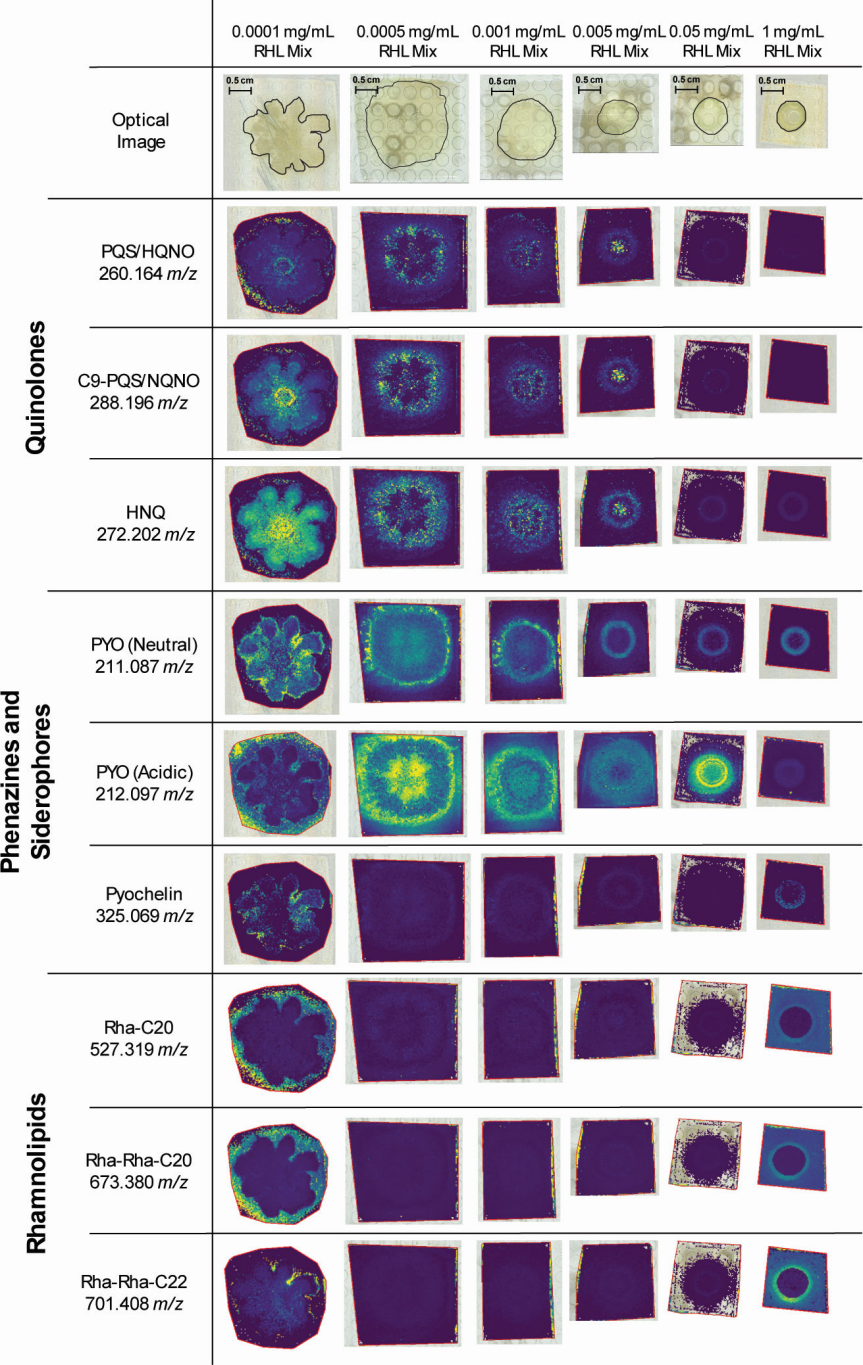
MSI analysis of swarming cultures in the presence of rhamnolipids. The yellow color indicates higher chemical abundance, while the blue color indicates lower chemical abundance in the image. With the increase in rhamnolipid concentration, we observe a loss of signal on most reported molecules. Scale bars (0.5 cm) can be found in the optical image of each condition. The 0.5% agar swarming condition in Fig. 3 was used to compare the spatial distribution of the metabolites under rhamnolipid conditions to a 1.5% agar control. Some images exhibit several points that lack MS information due to a technical issue during data acquisition.

Lastly, we wanted to explore if MSI could be used to monitor environmental changes for swarming studies. We investigated how the presence of rhamnolipids, generally positively associated with swarming, influences swarming characteristics. The addition of rhamnolipid to the swarming media can be categorized as a change in the growth environment, as previous studies have shown that rhamnolipids not only affect growth but also affect the secretion profile of *P. aeruginosa* (14, 43). To study *P. aeruginosa* swarming under different levels of rhamnolipids, a commercial rhamnolipid mixture was added to the M8 minimal medium agar at total concentrations ranging from 0.0001 to 1 mg/mL. Figure 4 shows the extracted ion images of *P. aeruginosa* metabolites. Interestingly, under 0.0001 mg/mL rhamnolipid mix, the swarming tendrils are more defined than those in the unmodified 0.5% agar swarming condition. Increasing the rhamnolipid mix concentration abolishes tendril formation, and at the highest rhamnolipid concentration, we observe a lack of surface expansion. With the loss of tendrils at higher rhamnolipid mix concentrations, we observe loss of signal or change in the location of secondary metabolite signals throughout the swarming culture, most predominantly the rhamnolipids. At 0.0001 mg/mL rhamnolipid mix, PQS is observed at higher abundance at the edge of the swarming culture on the agar surface, opposed to the center of the culture, as is observed in the swarming control. Another notable change is the abundance of PYO throughout the culture, most specifically observed on the 0.0005 mg/mL rhamnolipid mix conditions for acidic PYO. Tables 1 to 3 have an overview of all the reported secondary metabolites in this study and their presence under all reported conditions. A comparison between the culture conditions before and after drying can be observed in Fig. S2. The tendril differentiation seems to take place at the later stage of incubation and growth as the distinction is present after transfer and drying has taken place for the M8 medium control conditions absent of rhamnolipid mix and the 0.0001 mg/mL rhamnolipid mix conditions. To better understand if the presence of rhamnolipids affects bacterial growth, cultures in liquid swarming media were measured during the first 12 h and at 24 h. No exponential change in the optical density was observed under conditions of 0.05 mg/mL and lower (Fig. S1) as all of these conditions sustain a similar optical density throughout the collected time points. There was an observed increase in growth under liquid swarming conditions in the presence of 1 mg/mL of the rhamnolipid mixture (Fig. S3) when compared to the control and all other rhamnolipid mix concentrations. Overall, the external presence of rhamnolipids at 0.0001 mg/mL preserves the swarming morphology observed in control conditions and metabolic distribution patterns. However, concentration beyond 0.0001 mg/mL leads to significant changes, including increasingly smaller growth zones and the complete absence of swarming at higher concentrations. This does not affect bacterial growth under these conditions.

## DISCUSSION

Swarming is an important bacterial behavior that allows bacterial groups to translocate rapidly using flagella in semi-solid environments such as those found in the lungs. Previous studies suggest an important role of rhamnolipid as a wetting agent that enables swarming and colonization. However, contrasting observations highlighting the inhibitory effects of rhamnolipid on WT swarming when introduced at concentrations found in culture media have also been reported (10). However, the dose-dependent effect of rhamnolipids on swarming and tendril formation has not been thoroughly investigated. Swarming also requires collective behavior and thus involves several pathways that modulate quorum sensing and virulence factor secretion. Biomolecules such as quinolones, rhamnolipids, and phenazines are interconnected within the *P. aeruginosa* quorum sensing network (48), which leads to swarming and biofilm formation, inspiring us to investigate these molecules in this study. Delineating *P. aeruginosa* secondary metabolites production under different swarming conditions could enhance our fundamental understanding of metabolic pathways and potentially aid the discovery of novel drug targets that prevent further colonization. MSI is a powerful tool that can generate spatial chemical information and, in the context of *P. aeruginosa*, elucidate molecular changes during swarming and the influence of rhamnolipids. This technique has been used for co-infection studies as well (49), increasing its uses for therapeutic purposes. Compared to other methods like LC-MS or genetic reporters, MSI allows us to examine the culture as a whole without disturbing the bacterial sample, as observed in Fig. 3 and 4. The spatial estimation of these metabolites can be correlated to known or putative functionality, especially under different environments as observed in Fig. 4 in the presence of rhamnolipids.

Another advantage of using this technique to study secreted metabolites involves the feasibility of the sample preparation and the little perturbation to the natural state of the swarming culture, allowing us to study the bacterial environment directly opposed to other methods such as chemical extraction or cell lysis. The matrix application during the final steps of sample preparation can be used to derivatize any target molecules to enhance the signal of poorly ionizable molecules (50, 51). Other features can be used in tandem with MSI to improve the analytes observed on MSI, including trapped ion mobility spectrometry (tims), which is able to resolve isomers with the same molecular weight (i.e., PQS and HQNO in Table 2). MSI can be applied to bacterial swarming cultures in different chemical environments to study secretion and small molecule elucidation, including metabolites, lipids, peptides, and other biological molecules.

The reported secondary metabolites, quinolones, phenazines, and rhamnolipids, have been correlated to virulence factors (3, 24, 25, 52), although rhamnolipid function during infection is yet to be elucidated. The use of MSI on swarming cultures could lead to a better understanding of the functionality of these glycolipids or at least their spatial accumulation during swarming by rhamnolipid congener type. It can be inferred by our observations in Fig. 4 that Rha-Rha-C10-C10 is strongly associated with tendril formation on the agar surface, as we observe a loss of tendrils in the absence of any signal for this rhamnolipid. Using higher spatial resolution, MSI can aid in resolving the abundance of other rhamnolipids or secreted molecules found on the swarming surface.

Although MSI for bacterial cultures can be a useful tool, it does have its drawbacks. Firstly, our sample preparation, although seamless, does come with a few disadvantages. As each sample must be dried either through heat or forced air, real-time analysis of samples becomes difficult. A method to overcome this problem would be using liquid extraction surface analysis MSI, which has been a growing field for MSI analysis (33) or atmospheric pressure MALDI-MSI (53) and allows for intact colony analysis. Drying may also cause loss of signal for volatile molecules in the culture, preventing their identification and analysis. A loss signal can be compensated using derivatization agents prior to MSI analysis. Another drawback is the destruction of the samples due to the ionization laser used for MSI. Not only does drying the sample prevent further culture growth, but MSI analysis typically employs lasers as the ionization source, leading to sample burning and loss of signal post-analysis in the region of interest (ROI). A solution to this issue has yet to be found. Sample analysis becomes an issue with larger cultures as those similar to the control or the 0.0001 mg/mL rhamnolipid mix condition. Data acquisition on these samples takes between 6 and 10 h, depending on the sample area and image resolution, which dictates the number of points (pixels) of each image. Smaller samples such as we used with the 1 mg/mL rhamnolipid mix take 10× less time to acquire. The obvious statement is that lowering the spatial resolution reduces the acquisition time. A solution to large area acquisitions taking too long is to screen the entire agar plate at low spatial resolution and then select ROIs to acquire the higher spatial resolution detail. Finally, the ability to compare data taken over many months can be impacted by instrumentation effects. For example, in our case, changes to the instrumental response function caused by major maintenance affected the data collection of future samples. This effect can be observed in Fig. S4, where images observed in Fig. 1 are compared to swarming control conditions repeated 6 and 9 months later. Even with these effects, we detected the full complement of *P. aeruginosa* metabolites in our replicates (Fig. S4), demonstrating the robustness of the approach. A solution to such changing responses includes the use of robust batch correction approaches, although we suggest the simpler approach of preparing all required samples/replicates and analyzing them over a shorter time frame. Despite these drawbacks, MSI is still a faster, more streamlined method for surface analysis of bacterial cultures.

MSI analysis of *P. aeruginosa* swarming cultures can enhance our understanding of its swarming behavior and enable us to assess environmental parameters associated with infections that influence it. Here, we optimized culture conditions, sample preparation for MSI, and MSI data acquisition to profile important metabolite classes. The reported approach is robust and straightforward, allowing for sample preparation and analysis within the same day. It can be applied to a wide range of studies involving different swarming recipes, chemical agents, incubation protocols, and other parameters involving swarming. The applicability of this method will be important for further understanding swarming behavior in *P. aeruginosa* and other bacteria.

## MATERIALS AND METHODS

### Materials, strains, and media

Chemicals were procured from Fisher Scientific unless stated otherwise. PAO1C was used as the designated *P. aeruginosa* wild-type strain. LB media were prepared using the recipe provided by the manufacturer (Sigma Aldrich). LB agar plates were prepared using LB broth and 1.5%–1.8% Noble Agar (Sigma Aldrich). Swarming media were composed of a modified recipe of M8 minimal media (48 mM Na_2_HPO_4_, 24 mM KH_2_PO_4_, and 8 mM NaCl), 1 mM MgSO_4_, 0.5% Casamino acids (Sigma Aldrich), 0.2% glucose, and 0.5% agar.

### Preparation of swarming cultures and imaging plates

LB agar plates were prepared in 100 × 15 mm Petri dishes. All swarming conditions were prepared in 60 × 15 mm Petri dishes in triplicate. For MSI analysis, a replicate was chosen for MSI sample preparation. PAO1C glycerol stock was streaked on an LB agar plate and incubated at 37°C overnight. Subsequently, a liquid culture was prepared by transferring a colony from the streaked plate into a tube containing LB media and incubating it at 37°C overnight. For swarming plate inoculation, the overnight liquid culture was diluted to an optical density (OD_600_) of 0.3 in purified water using a Biochrom Ultrospec 10 Cell Density Meter (Harvard Bioscience, Holliston, MA). For all plates, 2 µL of diluted culture was inoculated in the center of the plate, and droplets were allowed to sufficiently dry to prevent any accidental dislocation. Once dried, the plates were incubated at 37°C for 18 h unless stated otherwise. Swarming agar was then transferred onto an MTP 384 target plate ground steel BC (Bruker Corp., Billerica, MA) and was dried at 37°C for 6 h or until dry. The transfer was performed by breaking the edges of the Petri dish and carefully sliding the agar without perturbing it onto the target plate using a spatula. Dried plates were stored at room temperature until matrix application. Liquid cultures were prepared using the M8 minimal media recipe, substituting the 0.5% agar volume with sterile water. All liquid cultures were inoculated with PAO1C overnight culture washed with freshly prepared M8 liquid media three times. Prior to incubation, all liquid cultures were adjusted to an OD_600_ of 0.01. Liquid cultures were prepared in four replicates and were incubated at 37°C for 24 h while shaking.

### MSI analysis

All samples were uniformly coated with 40 mg/mL of 2,5-dihydroxybenzoic acid matrix in 1:1 water:methanol using the overhead HTX M5 MALDI Matrix Sprayer (HTX Technologies, Chapel Hill, NC). The sprayer parameters were set to the following values: nozzle height, 40 mm; nozzle temperature, 70°C; lateral nozzle speed, 1,000 mm; lateral spacing, 3 mm; matrix flow rate, 0.05 mL/min; nitrogen pressure, 10 psi. The sample tray was maintained at 35°C, and the matrix was applied in four passes, with a 10-s delay between passes to allow drying. Samples were stored at room temperature until analysis.

MSI was performed using a timsTOF fleX MALDI-2 trapped ion mobility quadrupole time-of-flight mass spectrometer (Bruker Corp., Billerica, MA). All samples were scanned using a flatbed document scanner at 2,400 DPI to generate sample images and facilitate MSI analysis. timsControl (Client version 5.1.2, Bruker) was used to set the mass spectrometry parameters. All data were collected in a positive ion mode with a scan range of 150–1,000 m/z and a laser size of 100 µm. The MSI ROIs were designated using FlexImaging (Version 7.4, Bruker Daltonics GmbH & Co. KG). All MSI data were analyzed using SCiLS lab (Version 2023a Pro). Ion profiles were extracted using a ±16 ppm mass-extraction window for each m/z and were normalized using the total ion count. Ion profile comparisons are all obtained from the same ROI on each sample. Due to a technical issue with our instrument not sampling properly, there are specific points in the images where no information is obtained. Because of this, there is no intensity observed across all m/z values at those specific locations. We have not removed such points, and they are visible in the images shown in Fig. 3 and 4.

## Data Availability

SCiLS MSI data files, images used in the figures, and the tables found in the manuscript are available from the Illinois Databank. The figures are labeled by figure and by their title on each figure set. The tables are in an MS Excel sheet with the corresponding contents. The SCiLS data files contain the processed MSI data for all images. All files in the corresponding SCiLS data file must be present to open the individual data file. The feature list used for MSI analysis should be saved on the attached bookmark. SCiLS files can only be opened with the Bruker SCiLS software. Location: Sweedler, Jonathan; Rosado Rosa, Joenisse M. (2025): Data for Insights on the Spatial Distribution of the *Pseudomonas aeruginosa* Biofilm Secondary Metabolites Under Swarming Motility-Inducing Conditions Using Mass Spectrometry Imaging. University of Illinois Urbana-Champaign. https://doi.org/10.13012/B2IDB-9950768_V1.

## SUPPLEMENTAL MATERIAL

Supplemental figures spectrum.01368-25-s0001.pdf Figures S1 to S4.

Graphical abstract spectrum.01368-25-s0002.tif Visual depiction of study.

ASM does not own the copyrights to Supplemental Material that may be linked to, or accessed through, an article. The authors have granted ASM a non-exclusive, world-wide license to publish the Supplemental Material files. Please contact the corresponding author directly for reuse.
