# Supplementary material for: Insights on the spatial distribution of the Pseudomonas aeruginosa secondary metabolites under swarming motility-inducing conditions using mass spectrometry imaging
Source: Microbiol Spectr. 2025 Nov 11;13(12):e01368-25. doi: 10.1128/spectrum.01368-25 (PMC12671094; doi:10.1128/spectrum.01368-25)
Supplement: Supplemental figures — Figures S1 to S4. [file spectrum.01368-25-s0001.pdf]

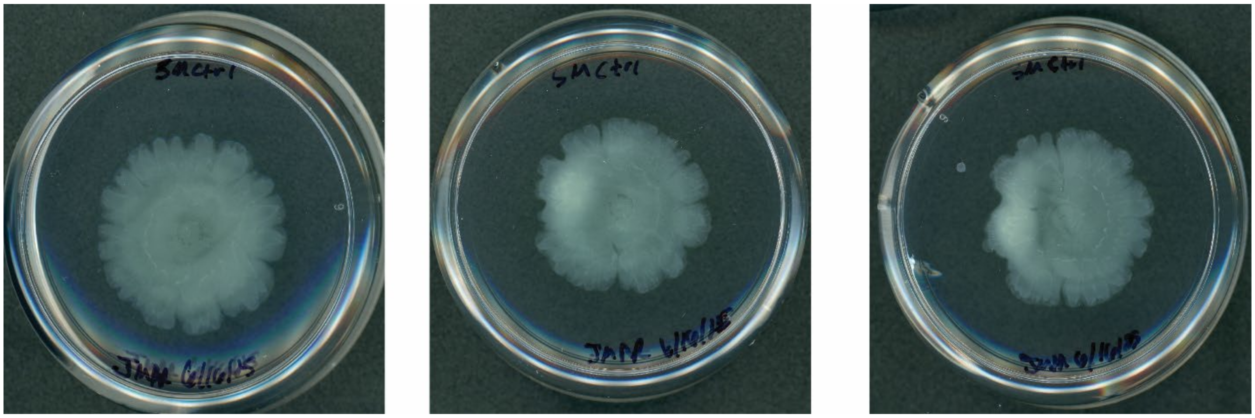

SI Figure 1: Control M8 minimal media swarming condition triplicate after 18 h incubation.

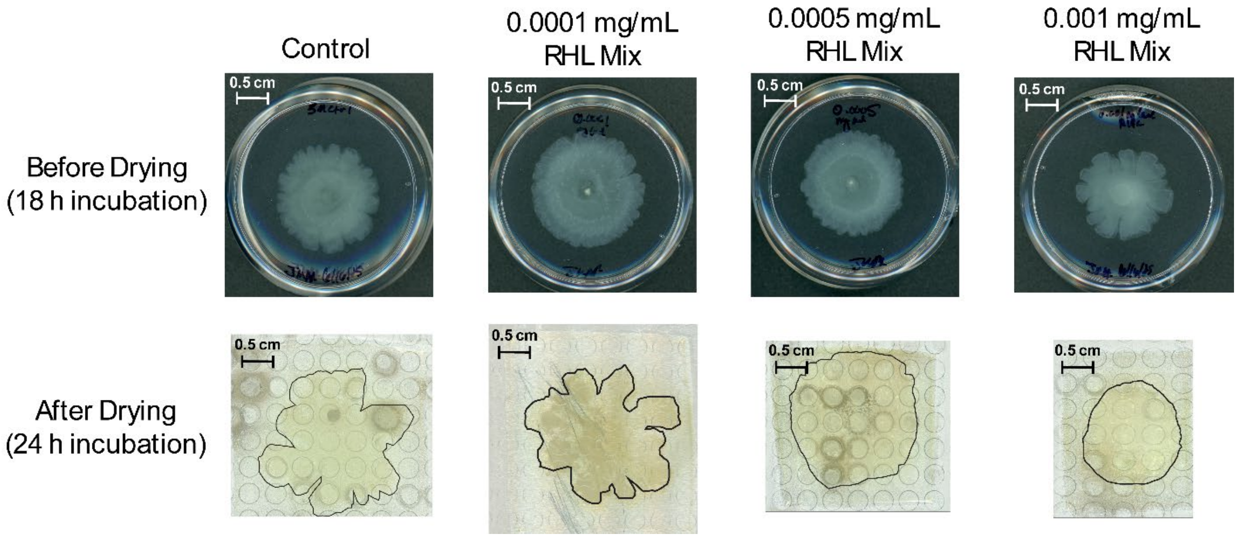

SI Figure 2: M8 minimal media growth effects before and after drying. All cultures are incubated for 18 h before transfer to MALDI target plate. The plates are then dried for 6 hours at 37 °C. The drying time allows for distinction of tendrils as is observed in the control and the 0.0001 mg/mL RHL mix conditions.

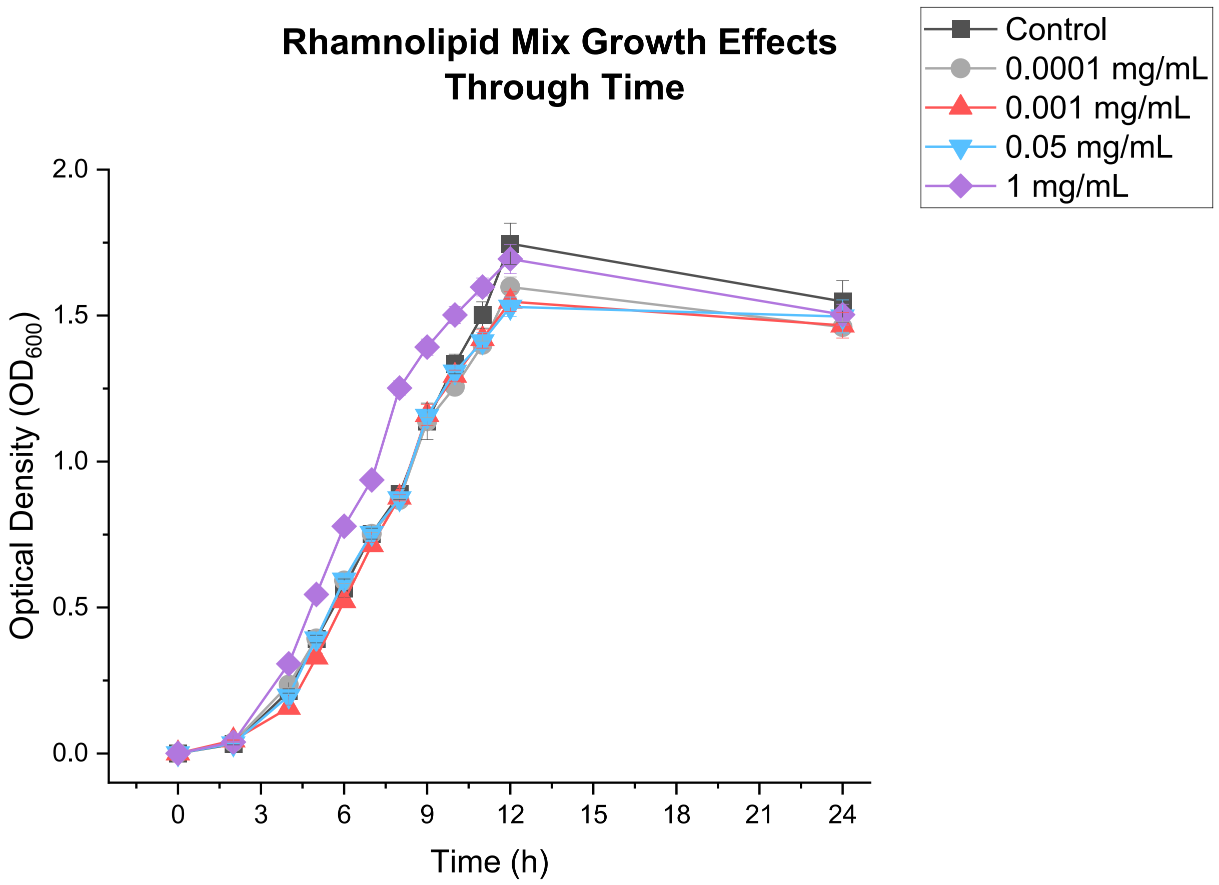

SI Figure 3: M8 minimal media liquid culture growth effects. Concentrations of rhamnolipid mix were added to M8 minimal media liquid cultures to assess growth through optical density measurements. Measurements were taken at 1-hour intervals, with the exception of timepoints at 2 and 4 hours. Optical density is measured from 2x dilution cultures. n = 5.

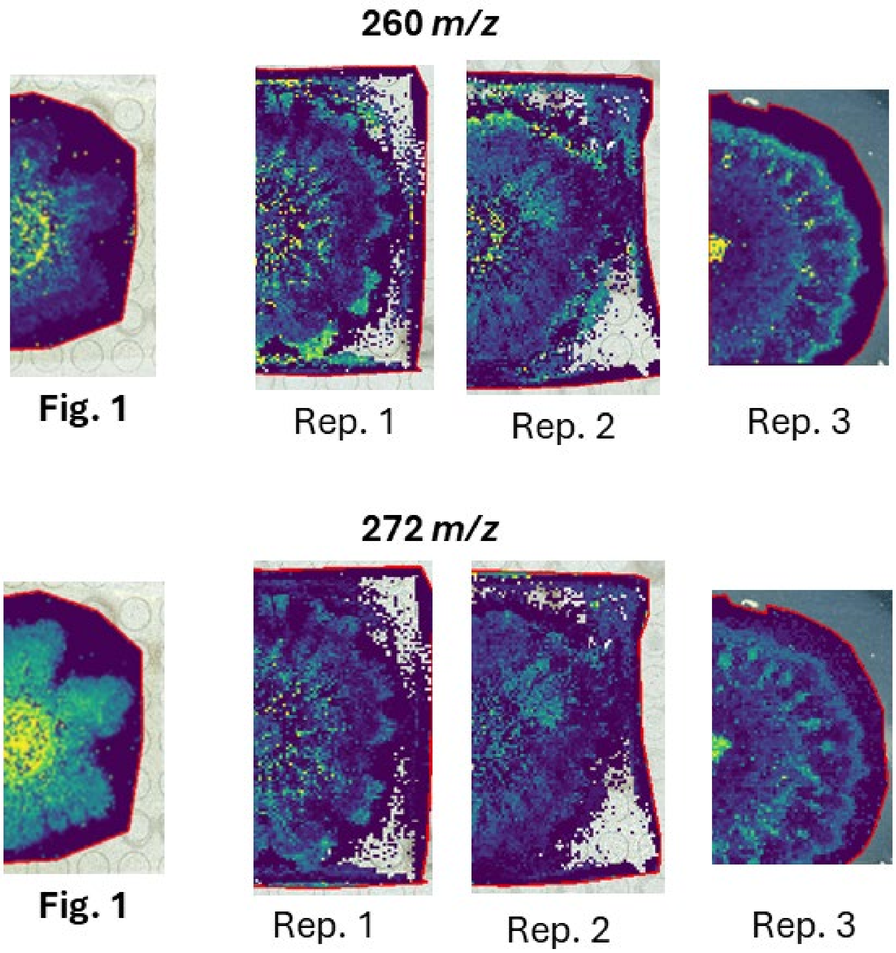

SI Figure 4: Replicates of swarming control conditions taken over 9 months. **Figure 1** images are the initial images, rep 1 and 2 were taken six months later and rep 3 taken 9 months after the initial image, with the common chemical and spatial features demonstrating the robust nature of approach. The white spots indicate a persistent issue with the instrument when operated in imaging mode but do not change the data quality at most locations.
