# Supplementary figures and images for: Insights on the spatial distribution of the Pseudomonas aeruginosa secondary metabolites under swarming motility-inducing conditions using mass spectrometry imaging
Source: Microbiol Spectr. 2025 Nov 11;13(12):e01368-25. doi: 10.1128/spectrum.01368-25 (PMC12671094; doi:10.1128/spectrum.01368-25)

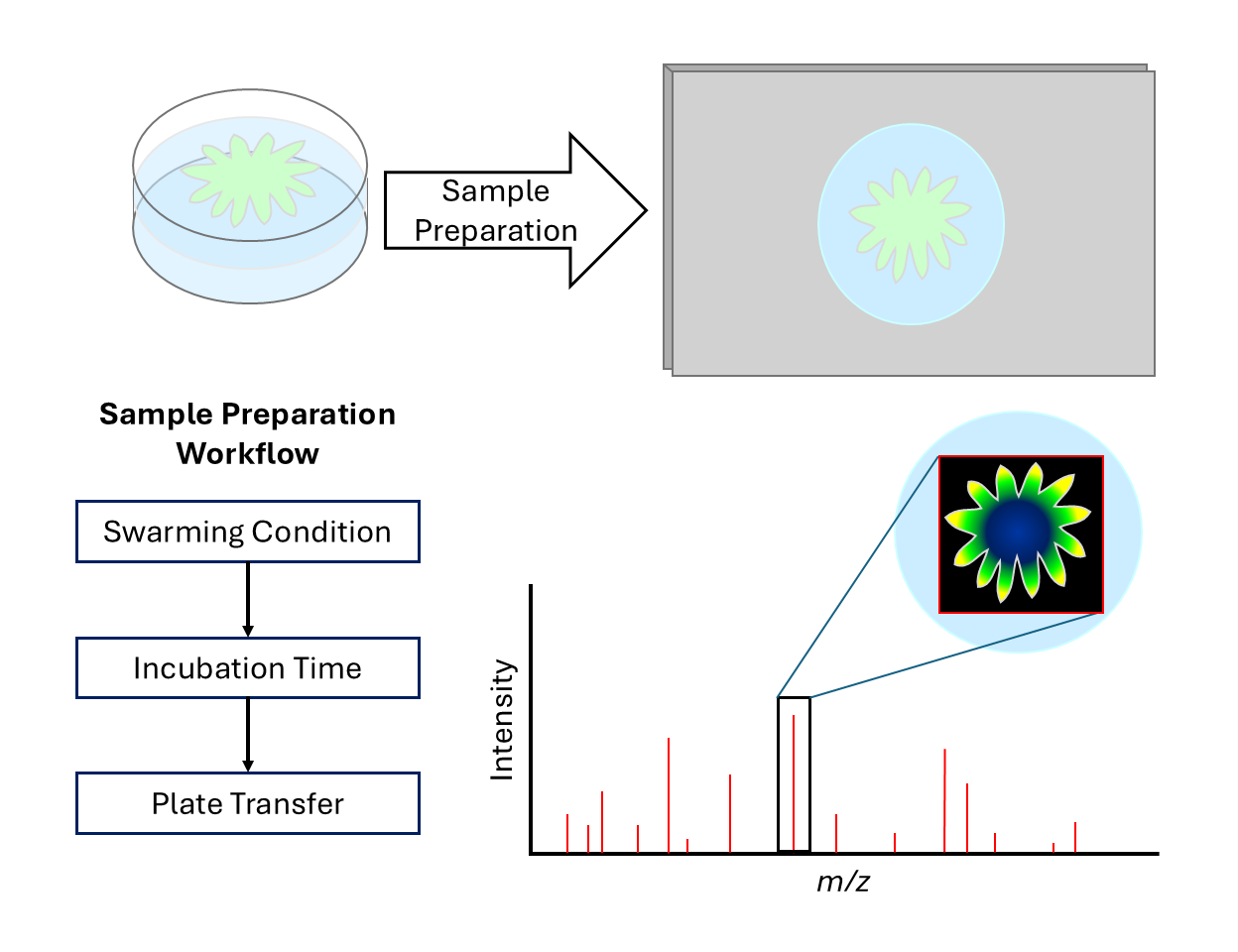

Supplement: Graphical abstract — Visual depiction of study. [file spectrum.01368-25-s0002.tif]
